# Supplementary material for: Metagenomics-Guided Survey, Isolation, and Characterization of Uranium Resistant Microbiota from the Savannah River Site, USA
Source: Genes (Basel). 2019 Apr 28;10(5):325. doi: 10.3390/genes10050325 (PMC6562407; doi:10.3390/genes10050325)

Metagenomics-guided Analysis, Isolation and Characterization of Uranium Resistant Microbiota Using a High-throughput Approach

Rajneesh Jaswal[1*], Ashish Pathak[1*], Bobby Edwards III[1], Robert Lewis III[1], John C. Seaman[2], Paul Stothard[3], Kirill Krivushin[3], Jochen Blom[4], Oliver Rupp[4], Ashvini Chauhan[1*]

[1]School of the Environment, 1515 S. MLK Blvd., Suite 305B, Building FSHSRC, Florida A&M University, Tallahassee, FL 32307, USA

[2]Savannah River Ecology Laboratory, The University of Georgia. PO Drawer E, Aiken, SC 29802, USA

[3]Department of Agricultural, Food and Nutritional Science, University of Alberta, Edmonton, AB T6G2P5, Canada

[4]Bioinformatics and Systems Biology, Justus-Liebig-University Giessen, 35392 Giessen, Germany

*Corresponding Author: Mailing address: Environmental Biotechnology Laboratory, School of the Environment, 1515 S. MLK Blvd., Suite 305B FSHSRC, Florida A&M University, Tallahassee, FL 32307, USA; Phone: 850-412-5119; Fax: 850-561-2248; E-mail: ashvini.chauhan@famu.edu

Table S1. Shotgun based sequence reads obtained from the SRS soils and DC/MT culture conditions. total Shown are sequencing depth, number of reads that mapped to taxonomic annotations, number of reads that mapped to a Phylum, and number of reads that mapped to the SEED functional database.

Sample ID	Raw Reads	Total NR (taxonomic) Alignments	Percent	Phylum Sum	Percent	Total Functional Alignments
DC Soil	9827394	2294597	0.23349	2066334	0.210263	1129787
MT Soil	2170172	457303	0.210722	411335	0.18954	225413
DC Gen 1	12630198	3518023	0.278541	3303629	0.261566	1597742
DC Gen 2	12206380	3027513	0.248027	2806697	0.229937	1386391
MT Gen 1	13656466	5622632	0.411719	5336589	0.390774	2782196
MT Gen 2	13852746	4002908	0.288961	3732288	0.269426	1894069

Table S2. Shown are the EDGAR identified set of gene homologues identified in strain MT2 that likely perform a biodegradative or metal resistance function(s)

Category	Gene Homologue
Metal Resistance Proteins	Metal tolerance protein 10<br>ATP-dependent zinc metalloprotease FtsH<br>Copper fist DNA binding domain protein<br>Iron transport multicopper oxidase FET3<br>Putative Zinc finger CCHC-type protein<br>Iron transport multicopper oxidase FET3<br>Siderophore iron transporter mirB<br>Iron-sulfur assembly protein 2<br>Magnesium transporter NIPA2
Transporter Proteins	Tetracycline transporter<br>General substrate transporter<br>CDR ABC transporter<br>ABC transporter substrate-binding protein<br>ABC transporter ATP-binding protein<br>Putative Monocarboxylate transporter
Cytochromes	Cytochrome P450 monooxygenase orf3<br>NADH-cytochrome b5 reductase 1<br>Cytochrome C biogenesis protein ResB
Drug Resistance	Multidrug resistance protein fnx1<br>Multidrug export protein EmrA
Efflux Pumps	Efflux pump FUB11<br>Efflux pump roqT<br>Efflux pump FUS6<br>Efflux pump dotC<br>Efflux pump dotC<br>Efflux pump FUB11

Figure S1. A, shown are the bacterial core microbiome at the genus level identified using shotgun metagenomic libraries from the SRS soils. Genera at 50% prevalence cutoff value was used to generate the plot. Also shown are the dendrogram analysis at the bacterial genus level using the Bray-Curtis index and the ward clustering algorithm (B). Groups shown in the plots refer to the metadata in which soils used in DC and MT experiments were binned: group 1 (soils used for DC/MT); group 2 (DC Gen1), group 3 (DC Gen2), group 4 (MT Gen1) and group 5 (MT Gen2), respectively.

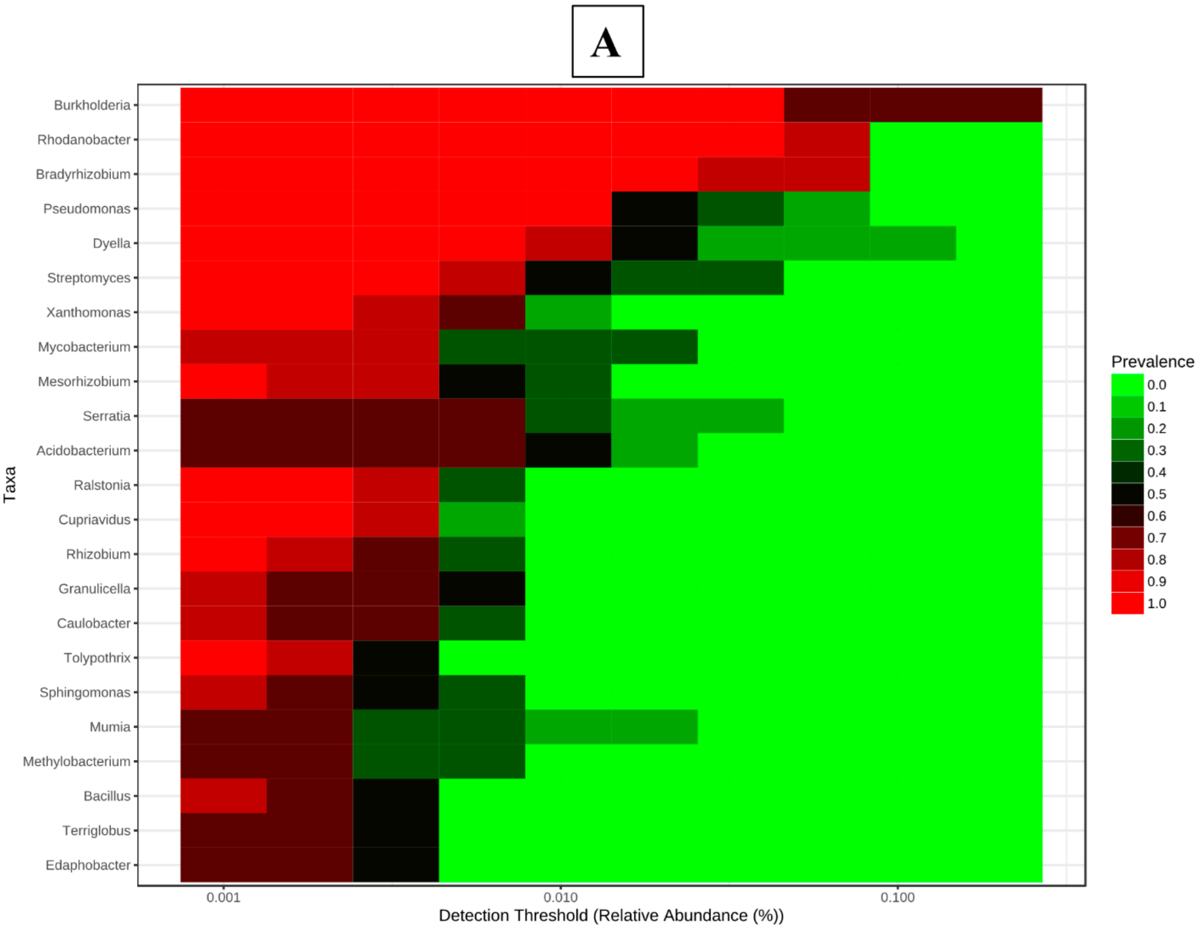

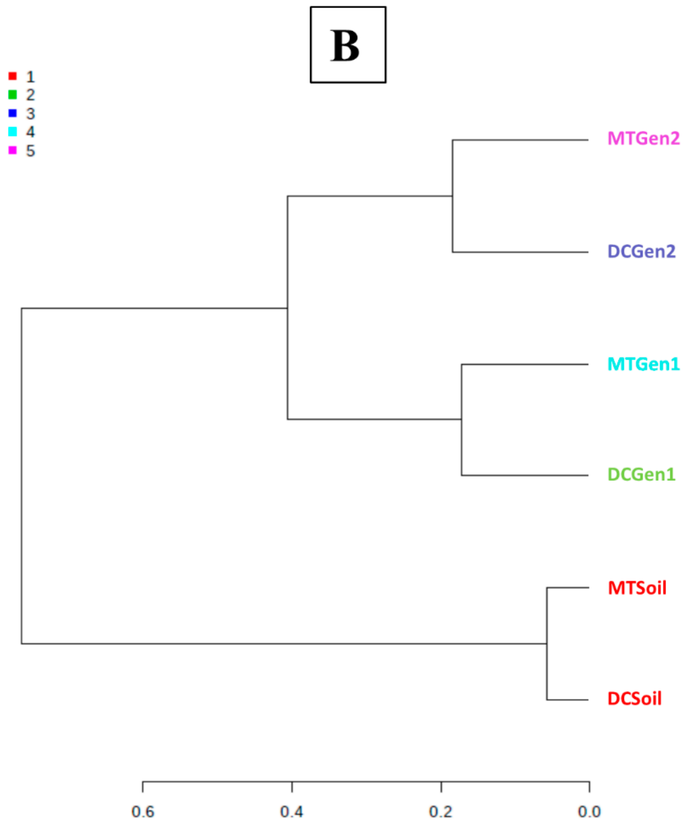
B
1
2
3
4
5
MTGen2
DCGen2
MTGen1
DCGen1
MTSoil
DCSoil
0.6
0.4
0.2
0.0

Figure S2. Shown are the (A), univariate statistical comparisons of *Burkholderia* spp., in soil (class 1) with Gen 1 DC/MT (class 2 and 3) and Gen 2 DC/MT (class 4 and 5) and (B), differential count analysis of *Burkholderia* spp., in soil (class 1) with Gen 1 DC/MT (class 2 and 3) and Gen 2 DC/MT (class 4 and 5).

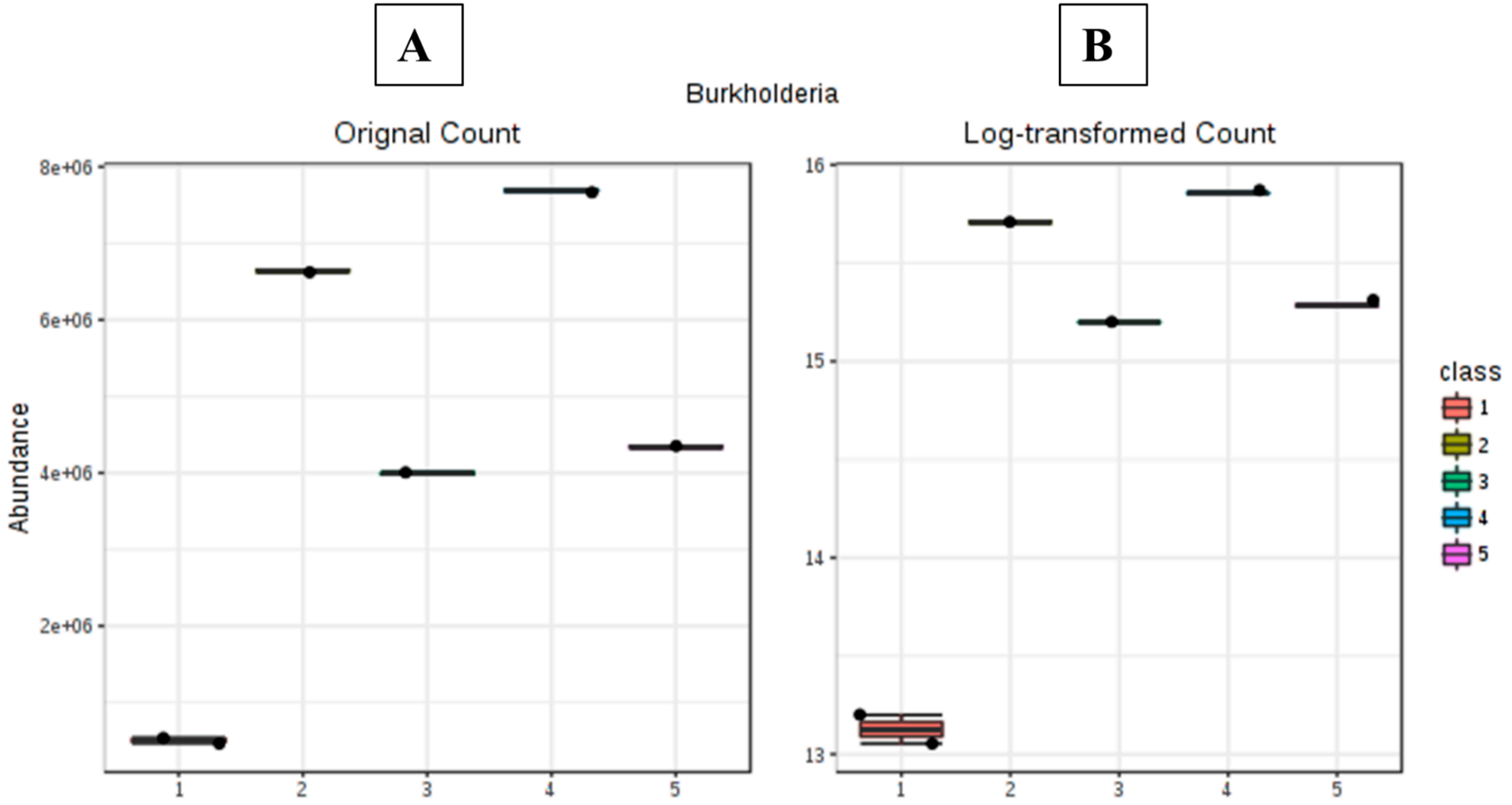

Figure S3. Archaeal relative abundance analysis shown at the phyla (A) and genus (B) levels identified from the soils and DC/MT chambers. Groups shown in the plots refer to the metadata in which soils used in DC and MT experiments were binned: group 1 (soils used for DC/MT); group 2 (DC Gen1), group 3 (DC Gen2), group 4 (MT Gen1) and group 5 (MT Gen2), respectively.

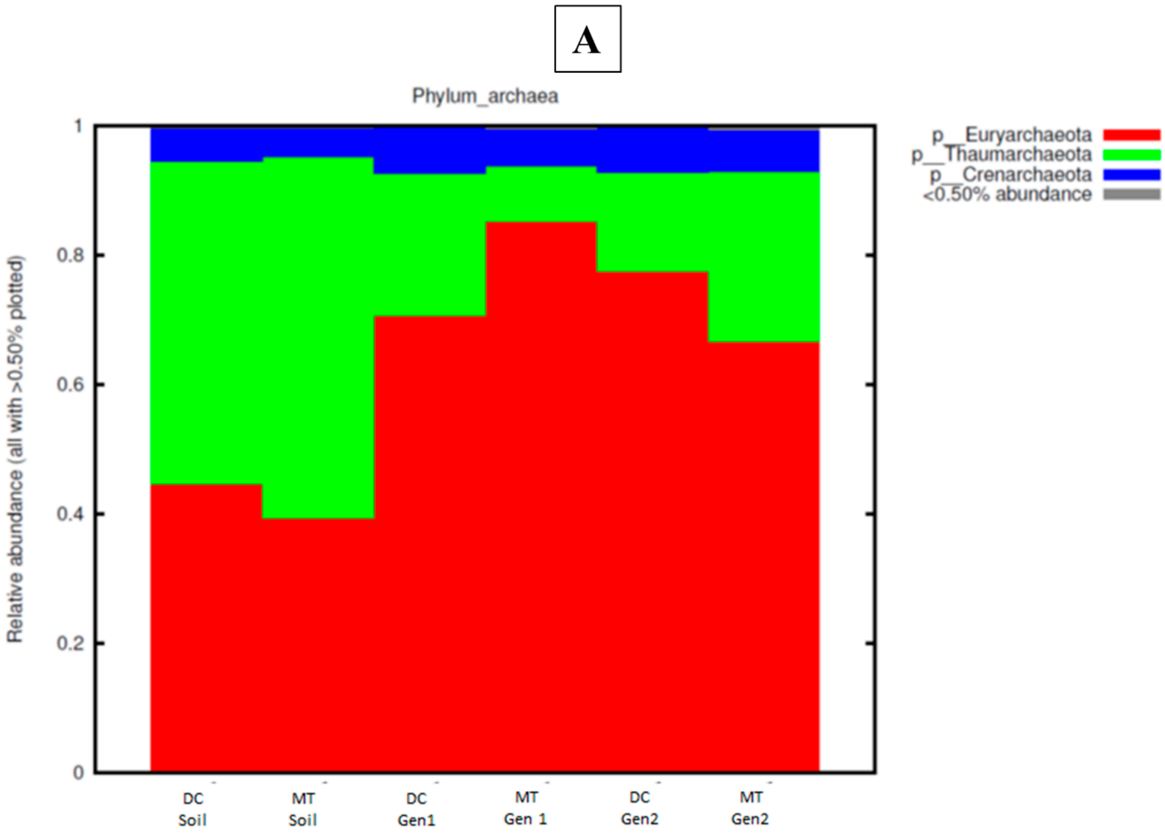

B

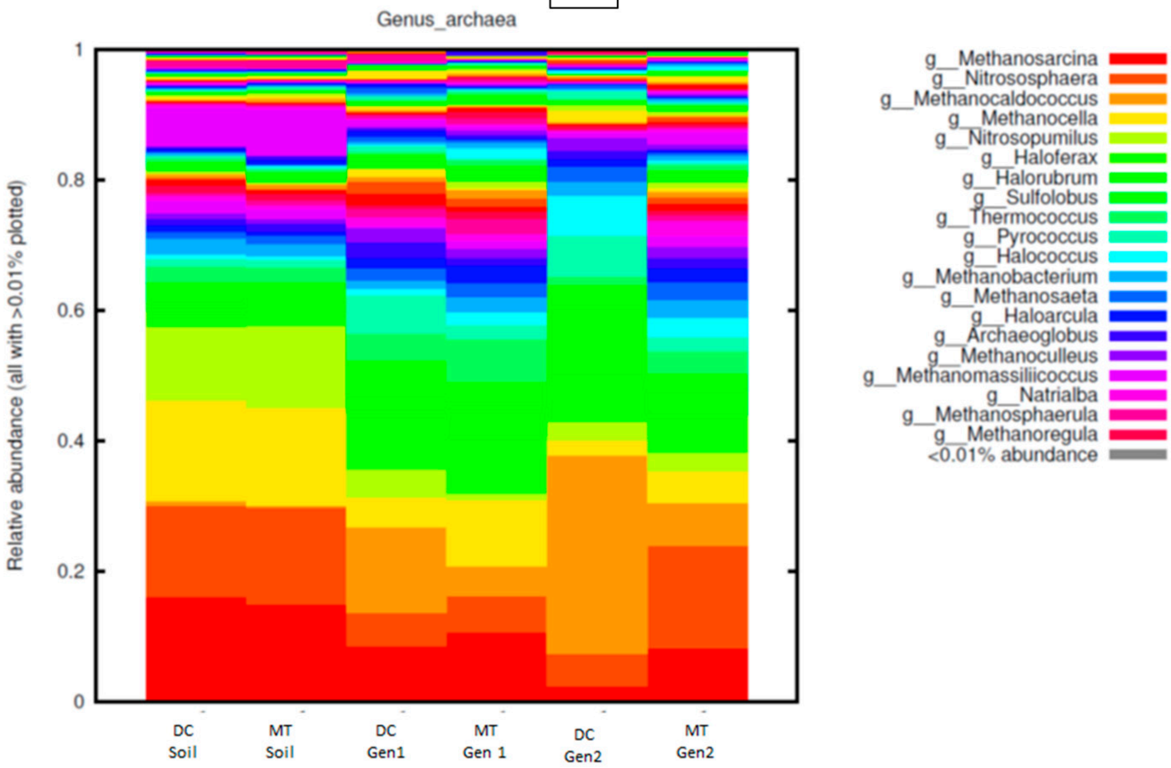
Genus_archaea
Relative abundance (all with >0.01% plotted)
0
0.2
0.4
0.6
0.8
1
DC Soil
MT Soil
DC Gen1
MT Gen 1
DC Gen2
MT Gen2
g__Methanosarcina
g__Nitrososphaera
g__Methanocaldococcus
g__Methanocella
g__Nitrosopumilus
g__Haloferax
g__Halorubrum
g__Sulfolobus
g__Thermococcus
g__Pyrococcus
g__Halococcus
g__Methanobacterium
g__Methanosaeta
g__Haloarcula
g__Archaeoglobus
g__Methanoculleus
g__Methanomassiliicoccus
g__Natrialba
g__Methanosphaerula
g__Methanoregula
<0.01% abundance

Figure S4. A, shown are the archaeal core microbiome at the genus level identified using shotgun metagenomic libraries from the SRS soils. Genera at 50% prevalence cutoff value was used to generate the plot. Also shown are the dendrogram analysis at the archaeal genus level using the Bray-Curtis index and the ward clustering algorithm (B). Groups shown in the figure refer to the metadata in which soils used in DC and MT experiments were binned: group 1 (soils used for DC/MT); group 2 (DC Gen1), group 3 (DC Gen2), group 4 (MT Gen1) and group 5 (MT Gen2), respectively.

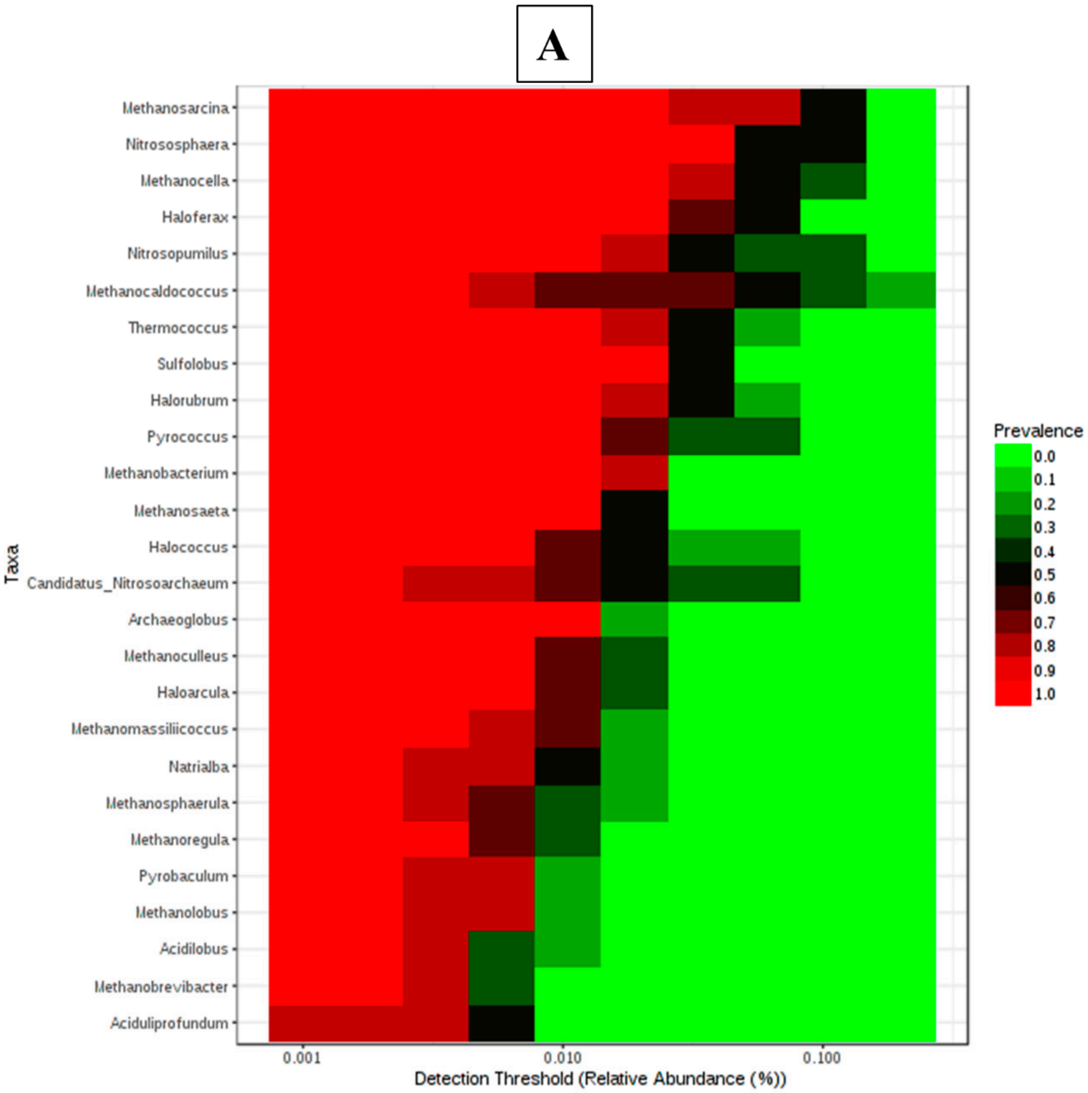

B

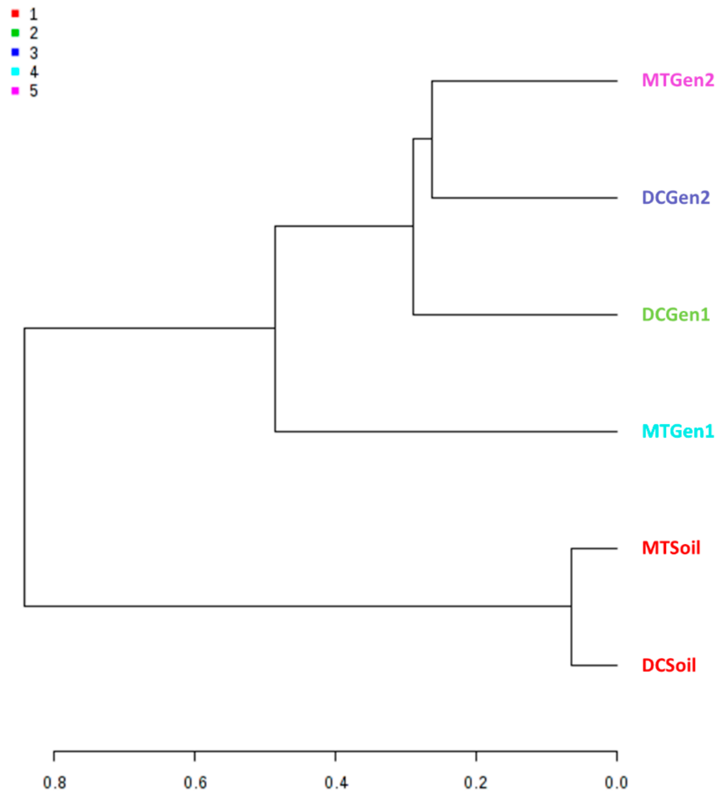
1
2
3
4
5
MTGen2
DCGen2
DCGen1
MTGen1
MTSoil
DCSoil
0.8
0.6
0.4
0.2
0.0

Figure S5. Shown is the heatmap analysis of gene functions identified from the SRS soils across different generations of DC/MT (A); the PCA statistical comparisons of gene functions identified in the SRS soils with different generations of DC/MT (B) and (C), the dendrogram analysis of gene functions using the unweighted UniFrac distance and ward clustering algorithm. Groups shown in the figure refer to the metadata in which soils used in DC and MT experiments were binned: group 1 (soils used for DC/MT); group 2 (DC Gen1), group 3 (DC Gen2), group 4 (MT Gen1) and group 5 (MT Gen2), respectively.

A

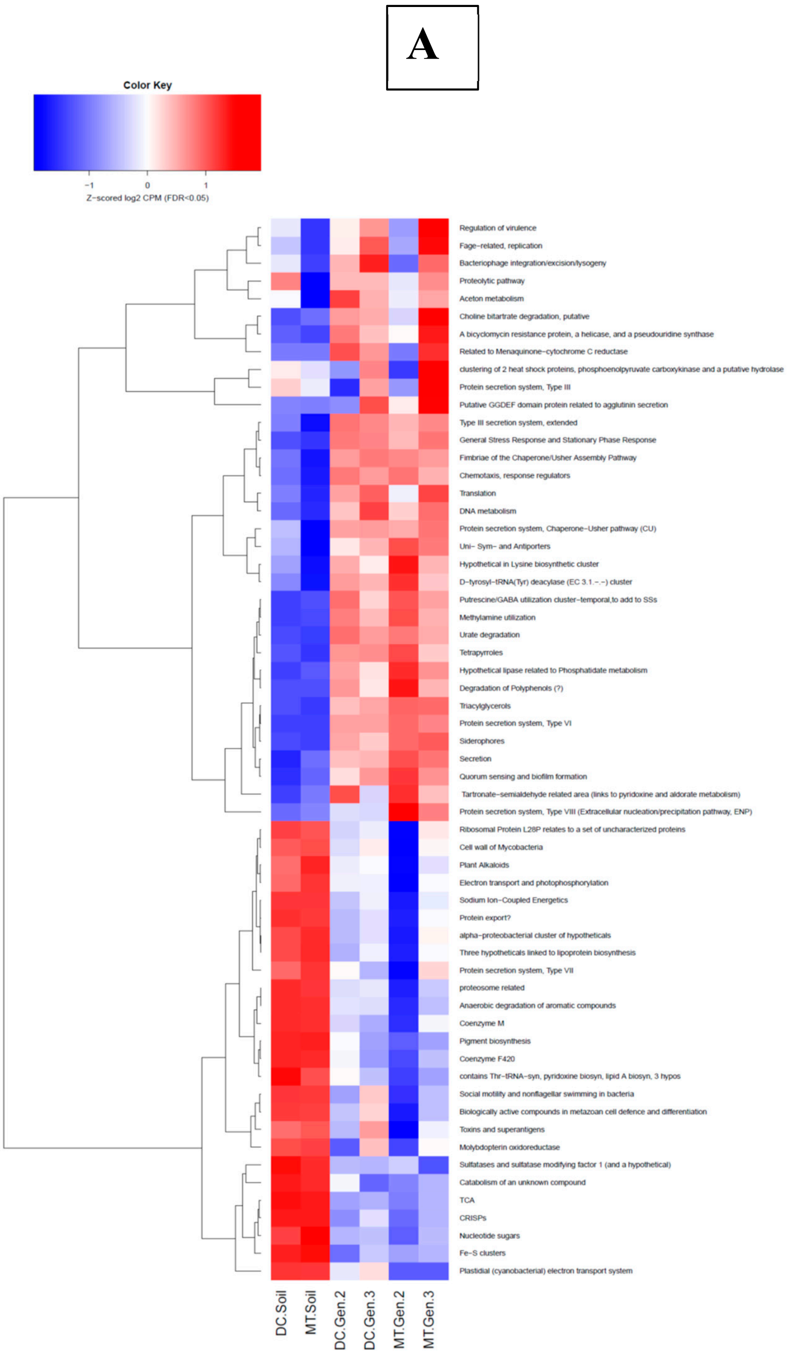

B

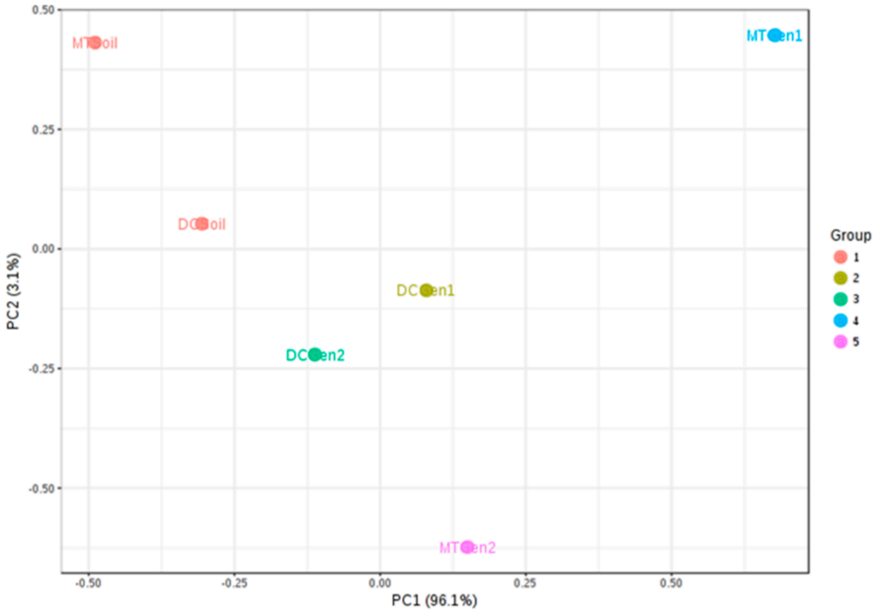

0.50
0.25
0.00
-0.25
-0.50
PC2 (3.1%)
-0.50
-0.25
0.00
0.25
0.50
PC1 (96.1%)
MTSoil
DCSoil
DCGen1
DCGen2
MTGen1
MTGen2
Group
1
2
3
4
5

C

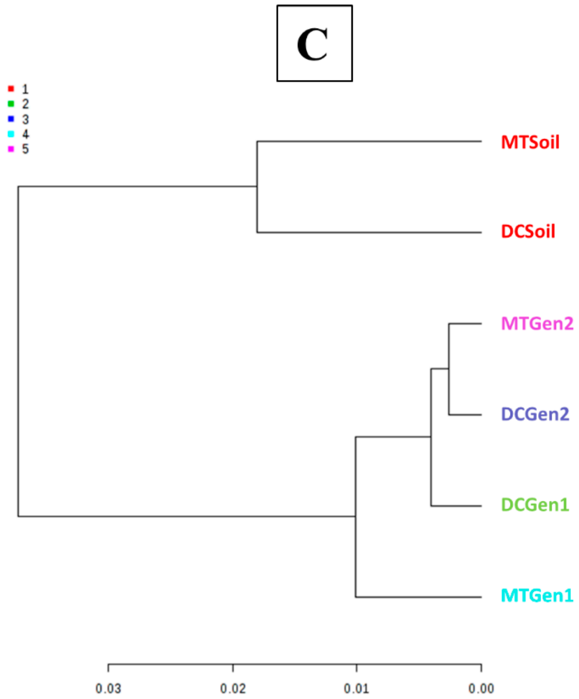

1
2
3
4
5
MTSoil
DCSoil
MTGen2
DCGen2
DCGen1
MTGen1
0.03
0.02
0.01
0.00

Figure S6. Shown is the 16S gene based phylogenetic tree of the isolated *Burkholderia* strains DC2 and DC3 relative to close taxonomic members (A). Also shown is the 18S gene based phylogenetic tree of isolated *Penicillium* strains DC2, DC3 and MT 2, respectively. The trees were constructed using neighbor joining bootstrap analysis in MEGA.

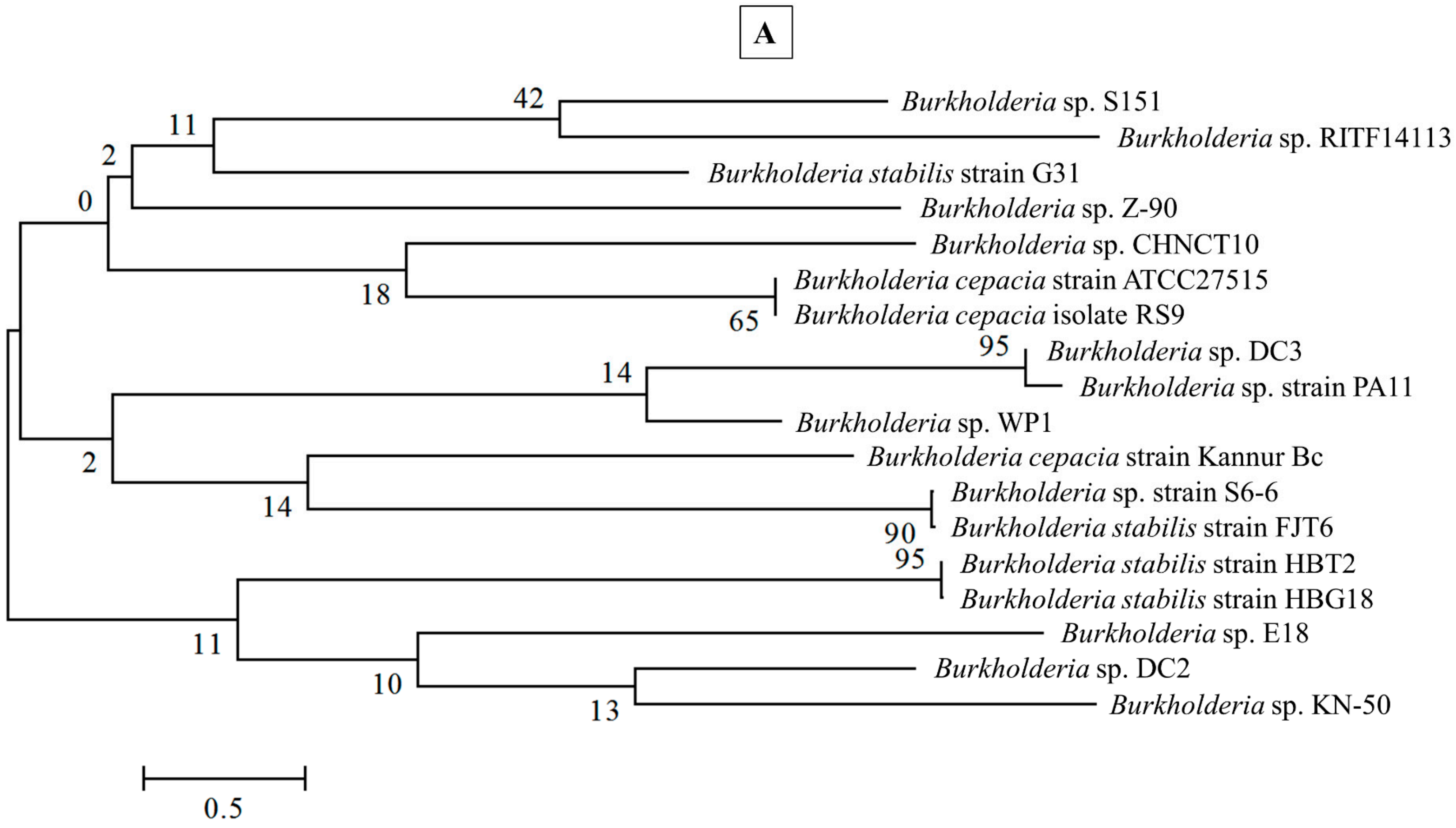

B

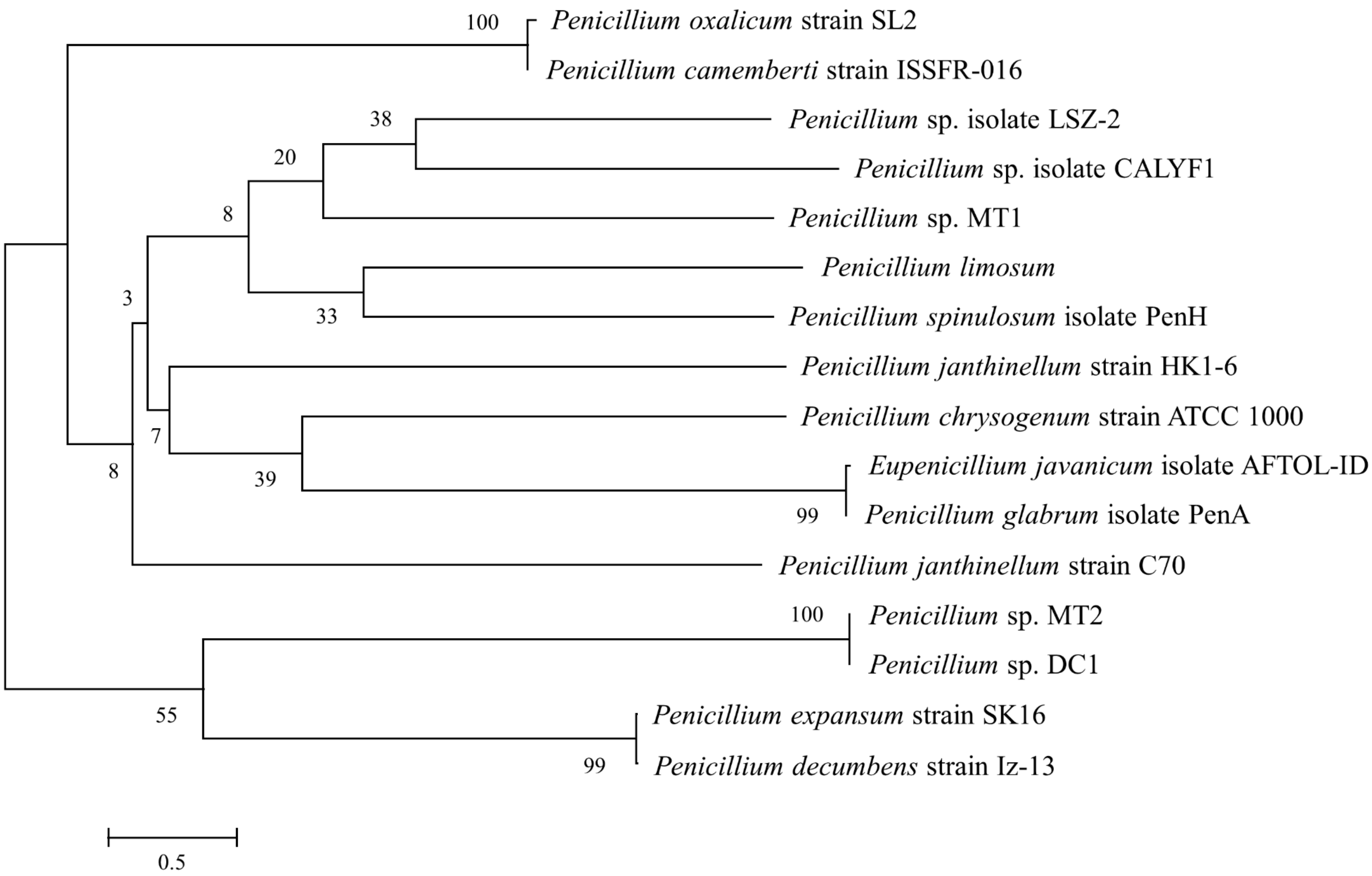
100
Penicillium oxalicum strain SL2
Penicillium camemberti strain ISSFR-016
38
Penicillium sp. isolate LSZ-2
20
Penicillium sp. isolate CALYF1
8
Penicillium sp. MT1
Penicillium limosum
3
33
Penicillium spinulosum isolate PenH
Penicillium janthinellum strain HK1-6
Penicillium chrysogenum strain ATCC 1000
7
8
39
Eupenicillium javanicum isolate AFTOL-ID
99
Penicillium glabrum isolate PenA
Penicillium janthinellum strain C70
100
Penicillium sp. MT2
Penicillium sp. DC1
55
Penicillium expansum strain SK16
99
Penicillium decumbens strain Iz-13
0.5

Figure S7. Whole genome sequence based synteny dot plots generated between *Penicillium sp. strain MT2* and the closest phylogenetic neighbor *P. janthinellum* strain NCIMI366.

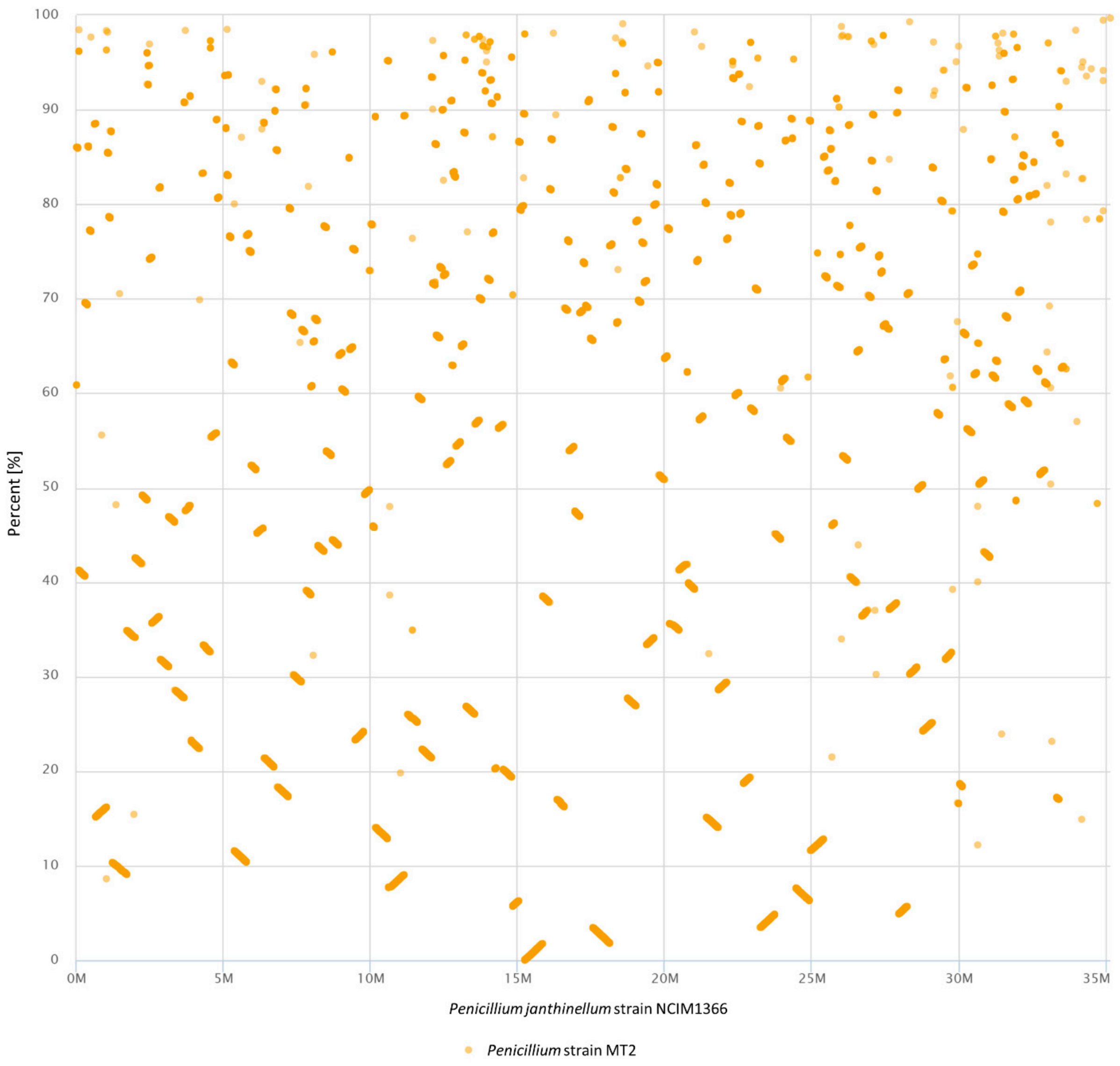

Supplement: Supplementary file 1 [file genes-10-00325-s001.pdf]
